# Fecal microbiota transplantation from female donors restores gut permeability and reduces liver injury and inflammation in middle-aged male mice exposed to alcohol

**DOI:** 10.3389/fnut.2024.1393014

**Published:** 2024-04-18

**Authors:** Arantza Lamas-Paz, Mariana Mesquita, Marcos Garcia-Lacarte, Olga Estévez-Vázquez, Raquel Benedé-Ubieto, Alejandro H. Gutierrez, Hanghang Wu, Hector Leal Lasalle, Javier Vaquero, Rafael Bañares, Eduardo Martínez-Naves, Sergio Roa, Yulia A. Nevzorova, Gonzalo Jorquera, Francisco Javier Cubero

**Affiliations:** ^1^Department of Immunology, Ophthalmology and Eye Nose and Throat (ENT), Complutense University School of Medicine, Madrid, Spain; ^2^12 de Octubre Health Research Institute (imas12), Madrid, Spain; ^3^State University of Campinas, Campinas, SP, Brazil; ^4^Department of Biochemistry and Genetics, Universidad de Navarra, Pamplona, Spain; ^5^Cancer Center Clínica Universidad de Navarra, Pamplona, Spain; ^6^Navarra Institute for Health Research (IdiSNA), Pamplona, Spain; ^7^Servicio de Aparato Digestivo, Hospital General Universitario Gregorio Marañón, Madrid, Spain; ^8^Instituto de Investigación Sanitaria Gregorio Marañón, Madrid, Spain; ^9^Centro de Investigación Biomédica en Red de Enfermedades Hepáticas y Digestivas, Madrid, Spain; ^10^Centro de Investigación Biomédica en Red de Cáncer (CIBERONC), Instituto de Salud Carlos III, Madrid, Spain; ^11^Centro de Neurobiología y Fisiopatología Integrativa (CENFI), Facultad de Ciencias, Universidad de Valparaíso, Valparaíso, Chile; ^12^Instituto de Nutrición y Tecnología de los Alimentos, Universidad de Chile, Santiago, Chile

**Keywords:** alcohol and gender, gut-liver axis, fecal microbiota transplantation, steatosis, senescence

## Abstract

**Background:**

Alcohol misuse, binge drinking pattern, and gender-specific effects in the middle-aged population has been clearly underestimated. In the present study, we focused on understanding gender-specific effects of alcohol exposure on the gut-liver axis and the role of gut microbiota in modulating gender-specific responses to alcohol consumption.

**Methods:**

Fifty-two-week-old female and male C57BL/6 mice were fasted for 12 h, and then administered a single oral dose of ethanol (EtOH) (6 g/kg). Controls were given a single dose of PBS. Animals were sacrificed 8 h later. Alternatively, fecal microbiota transplantation (FMT) was performed in 52-week-old male mice from female donors of the same age. Permeability of the large intestine (colon), gut microbiota, liver injury, and inflammation was thoroughly evaluated in all groups.

**Results:**

Middle-aged male mice exposed to EtOH showed a significant increase in gut permeability in the large intestine, evaluated by FITC-dextran assay and ZO-1, OCCLUDIN and MUCIN-2 immuno-staining, compared to PBS-treated animals, whilst female mice of the same age also increased their gut permeability, but displayed a partially maintained intestinal barrier integrity. Moreover, there was a significant up-regulation of TLRs and markers of hepatocellular injury, cell death (AST, TUNEL-positive cells) and lipid accumulation (ORO) in male mice after EtOH exposure. Interestingly, FMT from female donors to male mice reduced gut leakiness, modified gut microbiota composition, ameliorated liver injury and inflammation, TLR activation and the senescence phenotype of middle-aged mice.

**Conclusion:**

Our findings highlighted the relevance of gender in middle-aged individuals who are exposed to alcohol in the gut-liver axis. Moreover, our study revealed that gender-specific microbiota transplantation might be a plausible therapy in the management of alcohol-related disorders during aging.

## Introduction

Alcohol misuse is causing important morbidity and mortality worldwide (1). Globally, it causes 3.8% mortality and 4.6% of disability-adjusted life-years lost (2). Unfortunately, heavy drinking is increasing worldwide, and therapeutic management needs to be developed in order to fight this plague. According to the 2019 National Survey on Drug Use and Health, about 66 million, or about 24% of people in the United States ages 12 and older reported binge drinking (3). Binge drinking is typically defined by the National Institute on Alcohol Abuse and Alcoholism (NIAAA) as four of more drinks in adult women, or five or more drinks in adult men in a period of 2 h; with values of blood alcohol concentration (BAC) to 0.08% or higher (3).

Binge drinking became a common practice between young adults in developing countries; however, rates have been decreasing in the last decade, meanwhile the prevalence is increasing among older adults, with a past month prevalence- rate of 17.5% in middle-aged adults (4, 5). While the effects of binge drinking have been recently studied in young adults, the effects in middle-aged populations remain elusive (3). The high frequency of this practice and the lack of awareness in terms of the pathophysiological effects triggered by this form of alcohol misuse make it necessary to study.

Remarkably, alcohol disrupts the gut-liver axis, including alterations in the gut permeability [changes in tight junctions (TJs) and mucus barrier], gut microbiome dysbiosis (disturbances in the composition of the microbiome producing a disbalance of pathogenic and commensal organisms), and antimicrobial peptide production (increases microbial exposure to these peptides and development of a proinflammatory environment in the liver) (6–8). Current data indicate that besides the direct toxic effect of alcohol on liver parenchymal cells, abnormal gut microbiota, loss of intestinal barrier function, and the resultant activation of toll-like receptors (TLRs) on liver immune cells contribute to the pathogenesis of alcohol-related liver disease (ArLD). Notably, the contribution of an altered gut microbiota to ArLD begins before any indication of liver disease (9). Emerging evidence showed that gut microbiota composition is critical to maintain homeostasis of the gut-liver axis, and as part of this bidirectional communication the liver shapes intestinal microbial communities (9).

In the present study, we aimed to thoroughly examine the effects underlying the pathophysiology of alcohol intoxication in middle-aged female and male mice, evaluating the role of gender-specific gut microbiota and its influence on the gut-liver axis.

## Materials and methods

### Animal model of acute EtOH intoxication

C57BL/6J background from ENVIGO (Valencia, Spain) were bred and maintained in the animal facility of the Complutense School of Medicine in a temperature and humidity-controlled room with 12-h light/dark cycles and allowed food and water *ad libitum*. For our study, we used 52-week-old female and male mice; 52-week-old female mice are reproductively senescent (10). Animal studies were approved by the Conserjería de Medio Ambiente, Administración Local y Ordenación del Territorio (PROEX 125.1/20 and 264.2/23).

Acute administration of EtOH was done by oral gavage. In summary, wildtype (WT) mice (*n* = 4–11 per experiment) were fasted at night for 12 h. In the morning, they were fed with one oral dose of 30% EtOH (gavage of 6 g/kg body weight) with a gavage needle (Kent Scientific, Torrington CT) (11). As controls, we used phosphate-buffered saline (PBS) gavage. All animals were sacrificed with isoflurane (SOLVET, Segovia, Spain) 8 h after the challenge.

### Antibiotics treatment and fecal microbiota transplantation

Fifty-two weeks-old male mice were treated with a high-dose of an antibiotics cocktail (ABx) before FMT (day 1). ABx was prepared in sterile H_2_O at the following concentration: ampicillin (1 mg/ml), vancomycin (5 mg/ml), streptomycin (10 mg/ml), and metronidazole (10 mg/ml). this antibiotics cocktail combines b-lactam (ampicillin), glycopeptide (vancomycin), aminoglycoside (streptomycin) and nitroimidazole (metronidazole) antibiotics and was proved sufficient to decrease mouse gut microbial load within hours (12), targeting against the full spectrum of bacteria including both gram positive (ampicillin and vancomycin) and gram negative (ampicillin and neomycin) strains. All antibiotics were obtained from Gregorio Marañón University Hospital, Madrid (Spain). Antibiotics cocktails were freshly prepared on the day of treatment and was administered by one-time oral gavage (250 μl each mouse) on Day 0 after the collection of fecal samples.

Fecal pellets were collected from 52 weeks-old mice from all groups of mice before the ABx administration (Day 0). Stool from 52 weeks-old female mice were pooled, mixed and homogenized in PBS at concentration at 1 g feces/10 mL PBS (13, 14). Mixture was centrifuged at 500 rpm for 5 min at 4°C and supernatants were collected and used for FMT. Each 52 weeks-old male mice received 150 μL of the supernatant by oral gavage once a day continuously for 3 days (Days 2–4). Acute intoxication of 30% EtOH (gavage of 6 g/kg body weight) was administrated, and mice were sacrificed as previously mentioned 15 days after the FMT and at the age of 52 weeks-old (Day 19). EtOH gavage was performed as mentioned before (12). Stool from 52 weeks-old male mice was collected during and 16 days after FMT to further studies to analyze changes in gut microbiota.

### Histological and morphological analyses

Livers and colon from mice were harvested and after fixation with 4% PFA were embedded in paraffin for further histological evaluation. Hematoxylin and Eosin (H&E) and Oil Red O (ORO) staining were performed on liver and colon sections and liver sections, respectively as previously described (15, 16). Photomicrographs of stained sections were randomly taken in a 20X and 40X magnification in an optical microscope (Nikon Eclipse Ci, Tokyo, Japan) and Oil Red-O-positive areas were quantified using free NIH Image/J software (National Institutes of Health, Bethesda, MD, USA) as previously described (17).

### Biochemical measurements

Blood from the portal vein of each mouse was collected and sent to the University Hospital RWTH of Aachen, Germany and to Gregorio Marañon Research Institute (iISGM) in Madrid, Spain. Aspartate aminotransferase (AST) and alanine aminotransferase (ALT) were measured according to the standard procedures of the Central Laboratory Facility of the University Hospital RWTH of Aachen and Gregorio Marañon Research Institute as index of liver damage. Serum triglycerides were measured in samples in a collaboration with University Hospital RWTH of Aachen, Germany. For the evaluation of the intrahepatic triglyceride content, liver samples were homogenized in a specific Tris buffer (10 mM Tris, 2 mM EDTA, 0.25 M sucrose, and pH 7.5) and successively processed according to the manufacturer's instructions of a commercial colorimetric kit (10724600, Human Diagnostics).

### Quantitative real-time PCR

Total RNA was purified from liver tissue using Trizol reagent (Invitrogen, Karlsruhe, Germany). Total RNA (1 μg) was used to synthesize cDNA using Super Script first Strand Synthesis System (Invitrogen, Karlsruhe, Germany) and was resuspended in 100 μl of RNAse-free water (Sigma, St. Louis, MO, USA). Quantitative real-time PCR was performed using SYBR Green Reagent (Invitrogen, Karlsruhe, Germany) by the Genomics and Proteomics Facility (School of Biology, UCM). Relative gene expression was calculated using the 2^−ΔΔCt^ quantification formula normalizing each gene with the expression of GAPDH, using as an internal standard (18). Primer sequences can be provided upon request.

### Immunofluorescence and TUNEL assay

Liver and colon samples from each mouse were preserved in cassettes in Tissue-Teck (Sakura Finetek U.S.A, Torrance, CA) at −80°C. ZO-1, MUCIN-2, OCCLUDIN, F4/80, CD11b, β-GALACTOSIDASE and TUNEL tests were performed by standard procedures following the manufacturer's protocol (Roche, Rotkreuz, Switzerland).

### Analysis of intestinal permeability *in vivo*

Intestinal permeability was determined by measuring the appearance in blood of the marker isothiocyanate conjugated dextran (TdB Consultancy AB Uppsala, Sweden). Isothiocyanate conjugated dextran (FITC-dextran, molecular mass 4.0 kDa) (TdBCons, Uppsala, Sweden) was dissolved in PBS at a concentration of 200 mg/ml and administrated to 12 h fasted mice (10 ml/kg body weight) using a gavage needle (Kent Scientific). After 4 h, mice were sacrificed by an overdose of isoflurane (Solvet) inhalation. Concentration of FITC was determined in serum by fluorometry with an excitation of 485 nm and an emission wavelength of 528 nm using serially diluted FITC-dextran (0, 125, 250, 500, 1,000, 2,000, 4,000, 6,000, 8,000, 10,000 ng/ml) as standards.

### Microbiota analysis

Murine fecal samples were collected for bacterial DNA extraction using the centrifugal affinity column system in the PureLink^TM^ Microbiome DNA Purification Kit (Invitrogen), according to the manufacturer's instructions. Quantity and purity of the extracted genomic DNA were evaluated with a Thermo Scientific NanoDrop and stored at −20°C until used. For long-read sequencing of 16S rRNA gene, 10 ng of total genomic DNA were subjected to library construction using the 16S barcoding kit (SQK-16S024) according to Oxford Nanopore Technologies (ONT) protocol. Specifically, bacterial DNA was amplified using barcoded universal 16S primers (27F and 1492R) supplied by ONT with 5′-tags that facilitate the ligase-free attachment of rapid sequencing adapters required during nanopore library preparation. The barcoded libraries were loaded and sequenced on MinION flow cells (FLO-MIN106D R9.4.1; ONT) using the MinION-Mk1C instrument (ONT). After 24 h, a total of 398,221 nanopore reads in two pooled runs were obtained for the 11 individual samples of this study, with a median long-read length of 1.59 kb, consistent with the expected 16S rRNA size. Data acquisition, real-time analysis, base-calling, and data transmission of FASTQ files was operated using the MinKNOW (v22.10.7) operating software on the MinION-Mk1C sequencing device. The cloud-based EPI2ME Fastq 16S (v2023.04.21) algorithm was used for analytical workflow of 16S rRNA FASTQ files, including alignment and classification of reads into operational taxonomic units (OUT) to the species level, according to the NCBI 16S rRNA reference sequence database (containing over 26,000 curated type strain sequences of 16S rRNA from bacteria and archaea).

Read counts for mitochondria and chloroplast, as well as taxa with <5 observations in at least 10% of samples were filtered for downstream analysis using the Phyloseq R package (v1.38.0) (19), which was also used to calculate observed-OTUs and Shannon indexes for alpha diversity estimation. Finally, the MicrobiotaProcess R package (v1.15.0) (20) was used for generating rarefaction curves and further visualization of microbiota abundance and distribution across experimental groups.

### Statistical analysis

All statistical analyses consisted of One-Way ANOVA followed by Tukey *post-hoc* test using GraphPad Prism version 8.0.2 software (San Diego, CA). A *p* < 0.05 was considered statistically significant. Grubbs' Test to quantify outliers was performed using GraphPad. Data were expressed as mean ± standard error of the mean (SEM).

## Results

### Intestinal colonic permeability tends to be more resilient in middle-aged female mice, but not in same age male mice after acute EtOH gavage

**Figure 1:**
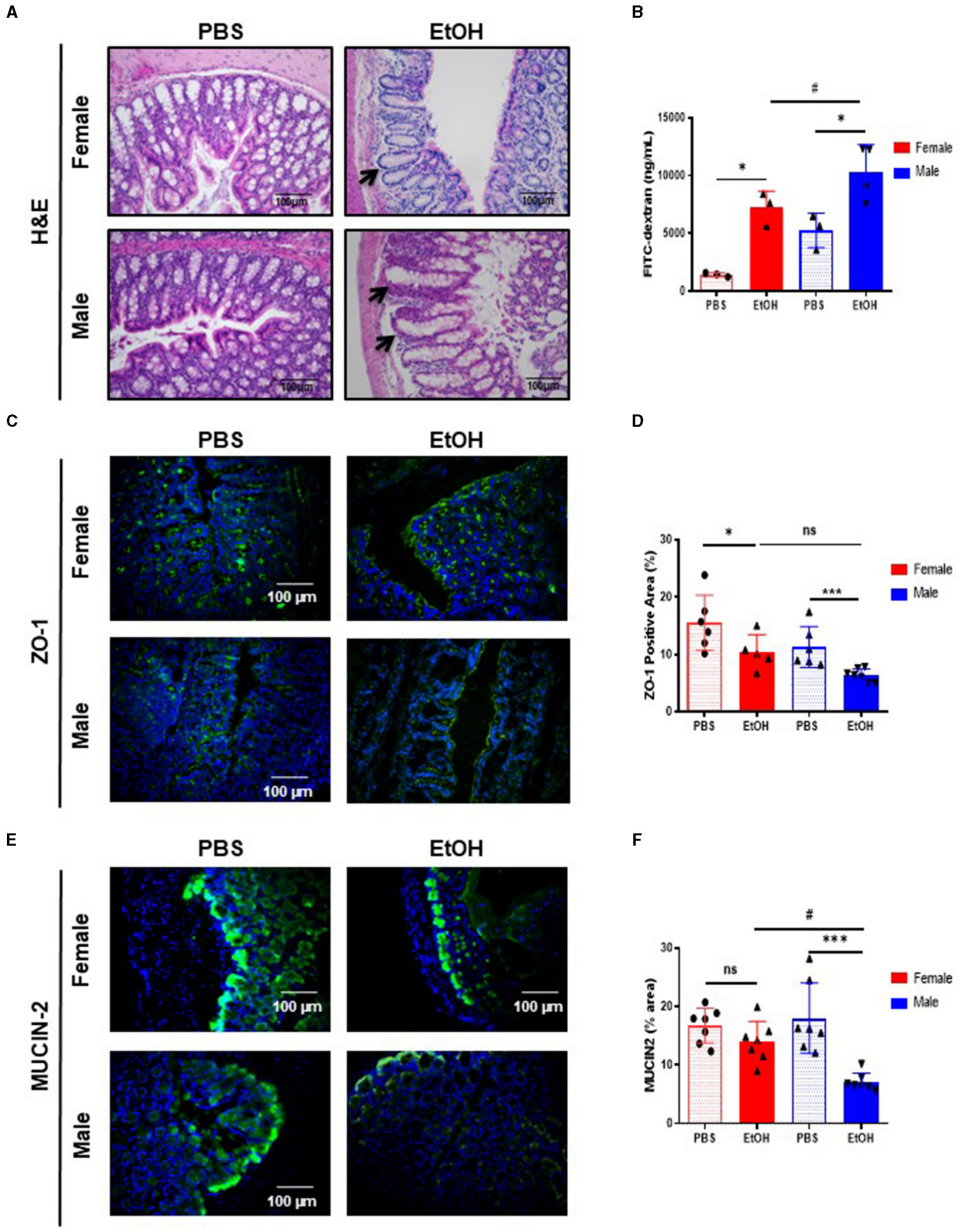
Intestinal barrier in large intestine of 52-week-old female C57BL/6 mice is partially maintained after acute ethanol exposure compared to same age male mice. **(A)** H&E staining of large intestine in 52-week-old female and male C57BL/6 mice treated with EtOH (6 g/kg) performed in paraffin liver sections. Crypt of Lieberkuhn is indicated by arrows. **(B)** Intestine permeability to FITC-dextran in female and male mice treated with EtOH/PBS gavage, represented as mg/mL serum (*n* = 3–4). **(C)** ZO-1 staining in large intestine of 52 weeks-old female and male mice after acute EtOH intoxication and **(D)** each quantification. **(E)** Mucin-2 immunofluorescence staining of 52 weeks-old C57BL/6 female and male mice treated with EtOH (6 g/kg) or PBS large intestine cryosections and **(F)** each quantification. (*n* = 7). Scale bars: 100 μm. *P < 0.05, ***P < 0.001, #*P* < 0.05. ns, no statistical difference.

In order to study the gender-dependent effects in the gut-liver axis after acute EtOH intoxication *in vivo*, 52-week-old female and male C57/BL6J mice were administered EtOH by gavage (6 g/kg) and we examined gut architecture and permeability. Interestingly, H&E staining of the large intestine showed that EtOH intoxication caused alterations in intestinal epithelium (changes in crypts) of 52-week-old male mice (Figure 1A); however, no relevant findings were observed in 52-week-old EtOH-fed female mice and PBS-gavaged mice (Figure 1A).

Since acute EtOH exposure could modulate gut permeability, we used the FITC-dextran method to determine intestinal permeability. Fifty-two-week-old female mice displayed significantly increased gut permeability, whereas same age male mice displayed exacerbated levels of FITC-dextran, compared to PBS-treated mice and even compared to EtOH-fed female (Figure 1B).

Intestinal permeability is associated with the integrity of the intestinal epithelial barrier. Intestinal epithelial tight junctions (TJs) acts as a gate, serving as a structural barrier against paracellular permeation of luminal antigens (21). TJs are composed of thin protein complexes, including zonula occludes-1 (ZO-1) and OCCLUDIN, that completely encircle the apex of the cell and make contact with TJs of adjacent cells, forming a continuous paracellular seal (22). Earlier we reported that acute EtOH intoxication disrupt TJs (23), thus we next evaluated whether changes in intestinal TJs, that result in leaky gut, occurred during experimental acute EtOH injury in 52-week-old female and male mice. Interestingly, the expression of ZO-1 is slightly but significantly decreased in the large intestine of 52-week-old female mice after EtOH intoxication, compared with PBS-gavaged female mice (Figures 1C, D). Interestingly, same age male mice show a dramatic decreased expression of ZO-1, compared with PBS-treated male mice (Figures 1C, D). Concomitant with these results, OCCLUDIN expression was significantly reduced in both groups of mice after EtOH intoxication, compared with PBS-treated mice (Supplementary Figures 1A, B).

The mucosa is covered by a thick layer of mucus that acts as a protective barrier against harmful substances, including bacteria. The major protein component of the intestinal mucous layer is MUCIN-2, which is secreted by Goblet cells and acts as the firs barrier that prevents direct contact between intestinal bacteria and colonic epithelial cells (24). Immunofluorescent staining revealed significantly lower expression of MUCIN-2 in the large intestine of EtOH-treated 52-week-old male mice compared with PBS-treated mice and statistically significant compared with same age female mice treated with ETOH (Figures 1E, F). Taken together, these results suggest that 52-week-old male mice displayed increased gut permeability, decreased number of TJs, and less mucous protection compared to female mice of the same age. However, female mice also exhibited gut disturbances, yet appeared to possess a more resilient gut physiology following experimental acute EtOH exposure.

### Fifty-two-week-old female mice display less liver damage after EtOH intoxication than male mice of the same age

**Figure 2:**
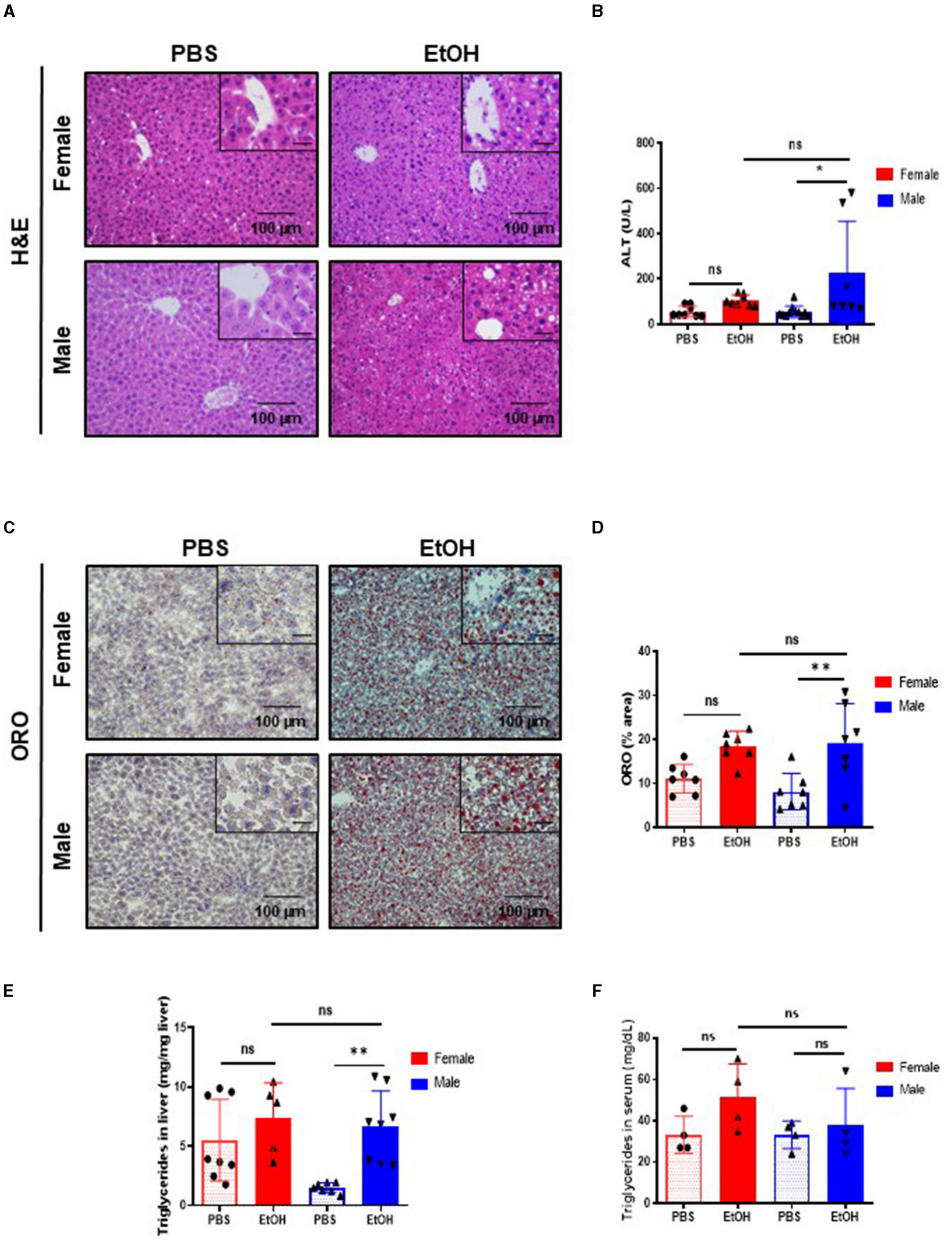
Fifty-two weeks-old female mice challenged with EtOH has less lipid accumulation in liver than same age male mice. **(A)** H&E staining in 52-week-old female and male C57BL/6 mice treated with EtOH (6 g/kg) performed in paraffin liver sections. **(B)** ALT levels measured in serum of 52-week-old female and male mice after EtOH or PBS (U/L) (*n* = 7–9). **(C)** ORO staining performed in liver cryosection of 52 weeks-old female and male mice liver treated with EtOH (6 g/kg) or PBS and **(D)** each quantification. **(E)** Quantification of triglyceride content in liver (mg TG/mg liver) in liver of 52 weeks-old female and male mice after an EtOH/PBS oral gavage. **(F)** Serum triglycerides (mg/dL) (*n* = 5–7). Scale bars: 100 μm. *P < 0.05, **P < 0.01. ns, no statistical difference.

Intestinal barrier dysfunction during EtOH exposure contributes to the development of liver injury (25). Since experimental acute EtOH exposure impaired intestinal barrier integrity, we subsequently investigated the effects in the liver. No macroscopic differences were found after acute EtOH intoxication in 52-week-old mice (Supplementary Figure 2A), albeit significant difference between female and male mice after EtOH in liver weight (LW) vs. body weight ratio (LW/BW) was found (Supplementary Figure 2B). Histological examination of livers by H&E staining revealed disorganized structure of the hepatic lobules in mice after acute EtOH exposure. The cytoplasm was translucent, exhibiting some extent of hepatocellular necrosis, and visible microsteatosis, especially in 52-week-old male mice. PBS-treated mice showed normal lobular architecture with sinusoidal hepatic cords and typical liver (Figure 2A).

Next, serum markers of liver damage were evaluated. ALT levels were slightly but significantly increased in 52-week-old male EtOH-treated mice, compared with EtOH-treated female or vehicle-treated mice (Figure 2B). Heavy alcohol exposure triggers hepatocyte cell death (26); thus, we studied cell death in the liver using TUNEL assay. The percentage of TUNEL-positive cells increased after acute EtOH exposure in 52-week-old male livers. A tendency toward increased cell death was observed in EtOH-fed female mice but no significance was found compared with same age male mice or PBS-treated animals (Supplementary Figures 2C, D).

Ethanol is metabolized in the liver by hepatocytes, the products derived from the metabolism of the EtOH leads to liver injury and hepatic steatosis (27–29). The staining of neutral lipids with ORO staining indicated a significant increase in lipid accumulation in livers of 52-week-old male mice challenged with EtOH compared to PBS-treated ones. Interestingly, same age female displayed no differences in lipid accumulation as observed by ORO staining (Figures 2C, D). In agreement with these observations, quantification of hepatic triglycerides showed a significant increase only in 52-week-old male mice after EtOH (Figure 2E), whereas serum triglycerides showed no differences between the experimental groups after EtOH intoxication (Figure 2F). In summary, our data suggested that, although middle-age female mice female experienced liver alterations after alcohol exposure, 52-week-old male mice were more susceptible to EtOH-induced hepatocellular injury and lipid accumulation.

### Fecal microbiota transplant from 52-week-old female mice donors prevented EtOH-induced gut dysfunction in middle-age male mice

To further investigate the role of the gut microbiota in the protection of the gut-liver axis after EtOH intoxication, we performed FMT in 52-week-old male mice. Fecal microbiota from healthy 52-week-old female donors was transferred by oral gavage during 3 days into same age male mice, which had been previously treated with an ABx cocktail the day before. Then, acute EtOH intoxication was performed by oral gavage (6 g/kg), 15 days after FMT as previously mentioned (11) (Supplementary Figure 3A).

**Figure 3:**
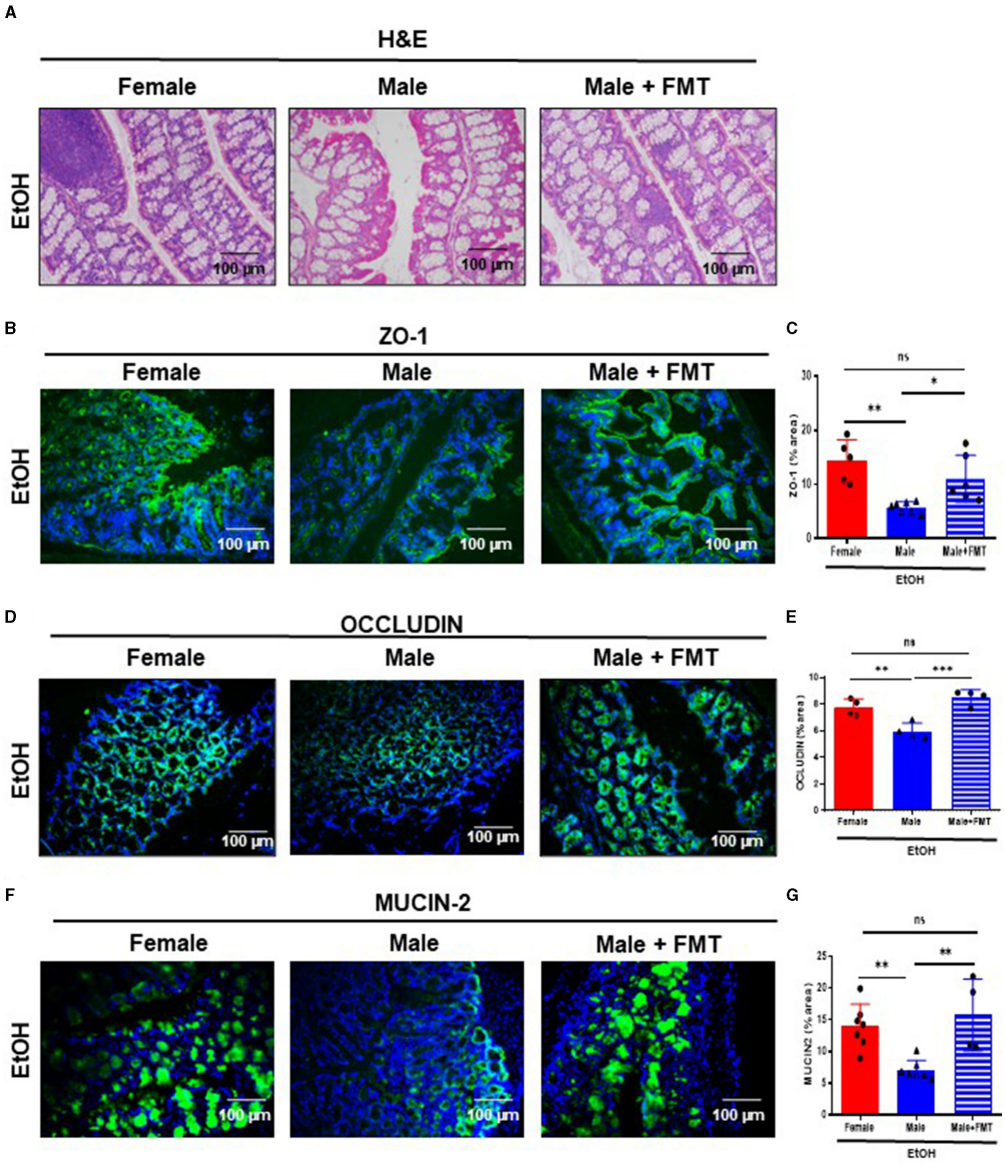
Fecal microbiota transplantation (FMT) from 52-week-old female donors reduces gut permeability in male mice of the same age after EtOH intoxication. **(A)** H&E staining of large intestine of 52-week-old female, male and male + FMT mice after EtOH oral gavage. **(B)** ZO-1 immunofluorescence staining in 52-week-old female, male, and male + FMT mice after EtOH gavage in large intestine cryosections **(C)** and each quantification. **(D)** Occludin immunofluorescence staining in large intestine cryosections of 52 weeks-old female, male, and male + FMT mice after EtOH (6 g/kg) oral gavage and **(E)** each quantification. (*n* = 4). Scale bars: 100 μm. **(F)** MUCIN-2 immunofluorescence staining was performed in large intestine of 52 weeks-old female, male, and male + FMT mice after EtOH oral gavage and **(G)** each quantification (*n* = 4–7). Scale bars: 100 μm. *P < 0.05, **P < 0.01, ***P < 0.001. ns, no statistical difference.

Interestingly, 52-week-old male mice after FMT + EtOH displayed improved intestinal barrier integrity similar to same age female mice. In fact, light microscopy examination of the large intestine of 52-week-old male + FMT mice revealed no relevant findings after EtOH intoxication (Figure 3A). Next, we evaluated gut permeability. We hypothesized that FMT to male mice would restore gut permeability to an extent similar to that observed in same age female mice. Interestingly, ZO-1 staining and quantification revealed significantly increased ZO-1 expression in TJs of 52-week-old male mice transplanted with female fecal microbiota and treated with EtOH, compared with non-transplanted male mice (Figures 3B, C). Concomitantly, FMT in 52-week-old male mice significantly elevated OCCLUDIN expression in the large intestine after EtOH exposure, compared with non-transplanted male mice and to the same levels as in same age female mice, treated with EtOH (Figures 3D, E). Moreover, immunofluorescent staining of MUCIN-2 in large intestine revealed similar values in 52-week-old male + FMT and female mice after EtOH, whereas, non-transplanted EtOH-fed male mice displayed a reduction in the mucus layer, as previously mentioned (Figures 3F, G). Altogether, gut microbiota from middle-aged female mice was capable of improved gut permeability function of same age male mice after EtOH exposure.

### Fecal microbiota transplantation in 52 weeks-old male mice after EtOH intoxication reduced hepatocellular injury and lipid accumulation

**Figure 4:**
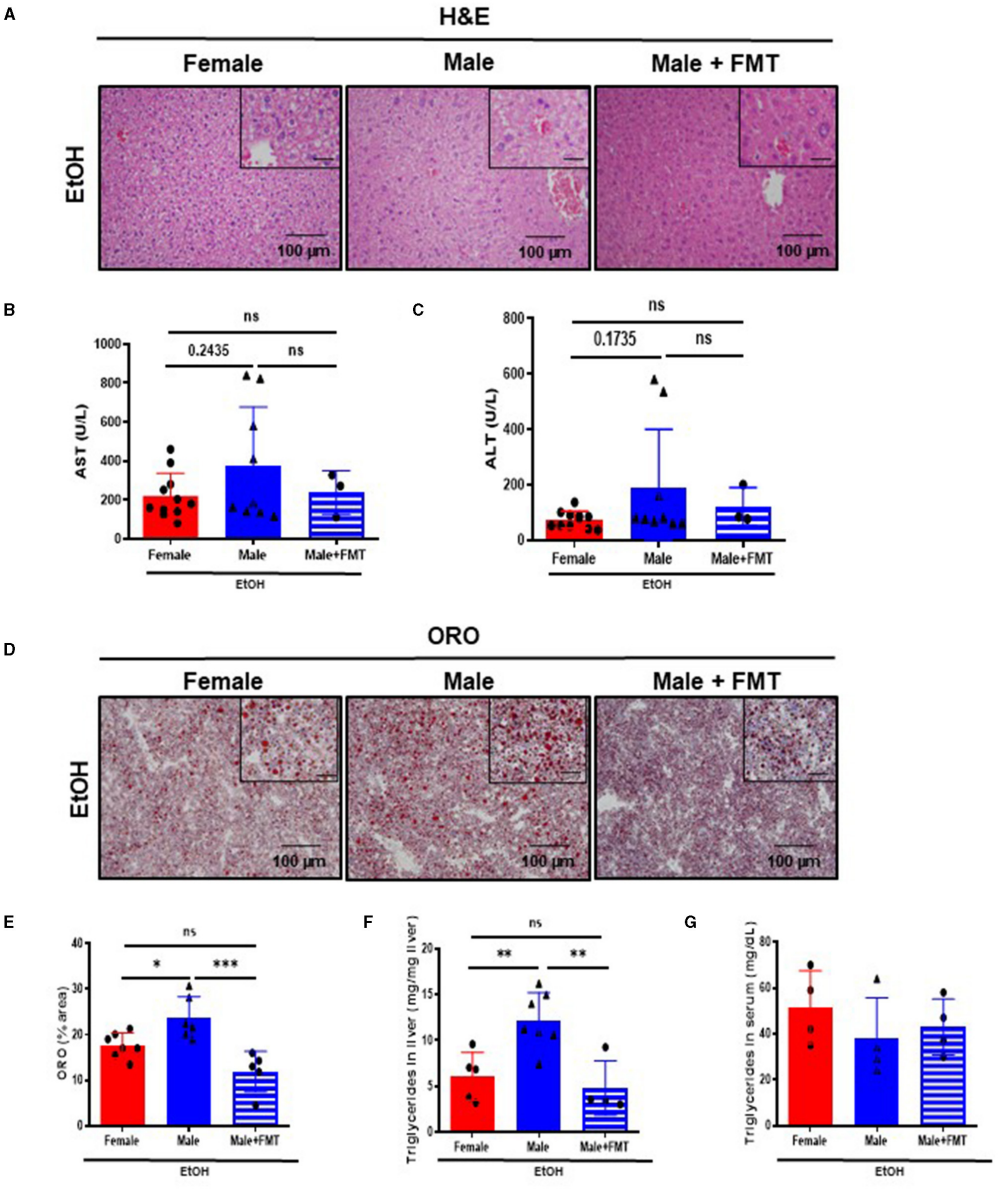
Fifty-two-week-old EtOH-fed male mice after fecal microbiota transplantation (FMT) display a protection against liver damage and lipid accumulation. **(A)** H&E staining of liver of 52-week-old female, male, and male + FMT mice after EtOH oral gavage. **(B)** Serum levels of markers of liver damage, aspartate aminotransferase (AST) and **(C)** alanine aminotransferase (ALT), in 52 weeks-old female, male, and male + FMT mice after EtOH (6 g/kg) oral gavage. **(D)** ORO staining in liver cryosections of female, male, and male + FMT mice after EtOH gavage and **(E)** each quantification. **(F)** Quantification of triglyceride content in liver (mg TG/mg liver) of 52-week-old female, male, and male + FMT mice after an EtOH oral gavage. **(G)** Serum triglycerides (mg/dL) (*n* = 4–11). Scale bars: 100 μm. *P < 0.05, **P < 0.01, ***P < 0.001. ns, no statistical difference.

Disruption of the gut permeability leads to microbiota translocation and liver damage. Macroscopic and microscopic examination of livers revealed no relevant findings in 52-week-old male mice + FMT after EtOH, compared with non-transplanted male mice (Supplementary Figure 3B; Figure 4A). Interestingly, serum markers of liver damage, such as AST and ALT, showed a tendency toward decreased levels in 52-week-old male mice + FMT after EtOH, with values similar to same age female animals (Figures 4B, C).

Additionally, lipid accumulation in liver was significantly reduced in 52-week-old male mice + FMT after EtOH. ORO staining showed a decrease in the total neutral lipids – quantified by % area – in the liver of 52-week-old EtOH-fed male + FMT mice (Figures 4D, E). In agreement with these results, hepatic triglyceride content in the liver measurement was significantly decreased in 52-week-old EtOH-fed male + FMT mice, compared with non-transplanted male mice (Figure 4F), albeit no differences were found in serum triglycerides in any of the experimental groups (Figure 4G). In summary, this set of data evidenced a role for FMT from middle-aged female donors in order to reduce hepatocellular injury and lipid accumulation as a result of EtOH intoxication in same age male mice.

### Fecal microbiota transplantation in 52-week-old male mice after EtOH intoxication ameliorated TLR cascade activation, immune cell infiltration, and activation of senescence

**Figure 5:**
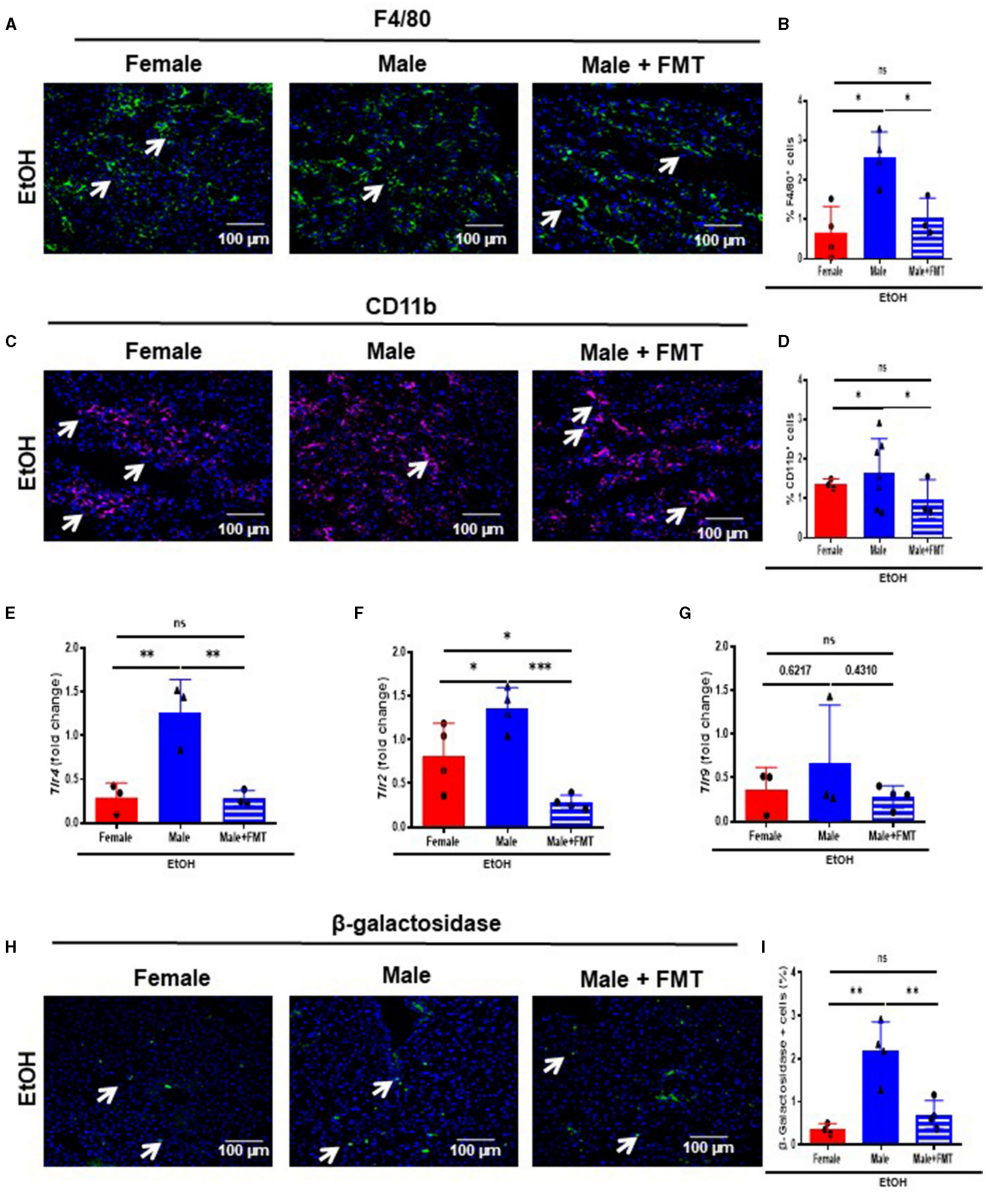
Fecal microbiota transplantation (FMT) reduces bacterial translocation, infiltration of immune cells and senescence after EtOH challenge in male mice. **(A)** F4/80 immunofluorescence staining in liver cryosections of 52-week-old female, male, and male + FMT mice after EtOH intoxication and **(B)** each quantification; and **(C)** CD11b immunofluorescence staining in liver and **(D)** each quantification. **(E)** *Tlr4*, **(F)** *Tlr2 and* **(G)** *Tlr9* mRNA relative expression to *Gapdh* in liver of 52 weeks-old female, male, and male + FMT after EtOH gavage **(H)** β-galactosidase immunofluorescent staining performed in liver cryosections of female, male, and male + FMT mice after EtOH oral gavage and **(I)** each quantification (% of positive cells) (*n* = 3–4). Scale bars: 100 μm. *P < 0.05, ^**^p < 0.01, ^***^p < 0.001. ns, no statistical difference.

We first evaluated immune cells infiltration into livers of our experimental groups. Therefore, we performed F4/80 and CD11b immunofluorescent staining which revealed a significant decrease in the percentage of F4/80- (Figures 5A, B) and CD11b- (Figures 5C, D) positive cells in 52-week-old male + FMT mice after EtOH, compared with non-transplanted male mice of the same age.

A decrease in the gut permeability reduces bacterial translocation and subsequently causes a decrease in the activation of Toll-like receptors (TLRs). Moreover, TLR4 triggers inflammation in alcohol-related liver disease (30). Thus, we first analyzed the mRNA transcripts of the TLR cascade. *Tlr4*, and *Tlr2* mRNA expression revealed a significant reduction in 52-week-old EtOH-fed male + FMT mice, compared with non-transplanted male mice of the same age (Figures 5E, F), with no significant changes in mRNA transcripts levels for *Tlr9* in 52-week-old EtOH-fed male + FMT mice (Figure 5G).

Inflammation and aging are closely linked with the activation of senescence-associated β-galactosidase (SA-β-GAL), which is associated to a senescent phenotype, contributing to cell cycle arrest and tissue disfunction (31). Therefore, we subsequently analyzed the expression of SA-β-GAL in our experimental groups. The percentage of SA-β-GAL-positive cells was significantly reduced in both 52-week-old female, and male + FMT mice after EtOH intoxication, compared to EtOH-fed male mice of the same age (Figures 5H, I). Altogether, FMT from 52-week-old female donors diminished bacterial translocation, immune cell infiltration and a senescence phenotype in the liver of same age male mice.

### Reconstitution of female-like gut microbiota in male mice treated with EtOH following FMT

**Figure 6:**
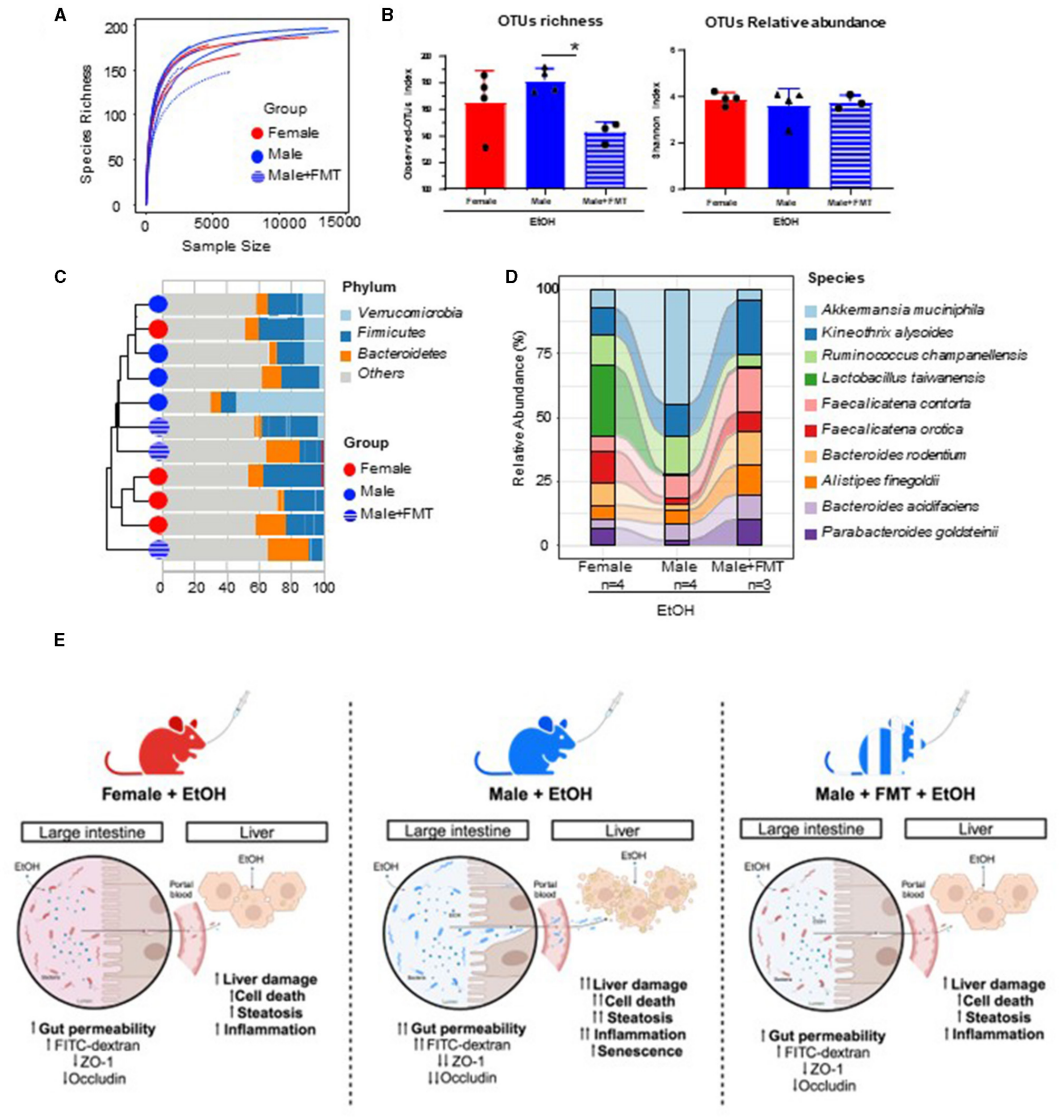
Characterization of changes in gut microbiota in FMT-male mice treated with EtOH. **(A)** Rarefaction curves after 24 h of nanopore-based long-read sequencing of bacterial 16S rRNA in feces from female, male, and male + FMT mice following EtOH treatment. **(B)** Alpha diversity as measured by Observed-OTUs and Shannon indexes across groups. **(C)** Hierarchical cluster analysis according to the distribution of phyla within the top 10 more abundant OTUs in each sample. **(D)** Sankey diagram showing the inferred relationship between groups by relative abundance (%) of top 10 classified OTUs at species level. **(E)** Proposed model for gender-specific pathophysiology of alcohol intoxication and the influence of female fecal microbiota transplantation (FMT) on the gut-liver axis of male recipient mice. ^*^p < 0.05.

To characterize changes in gut microbiota during FMT and EtOH treatments, we collected feces from age-matched female donors (*n* = 4), FMT male receptors (*n* = 3), and non-FMT male mice (*n* = 4) at the end of EtOH treatments. To evaluate changes in bacterial diversity among these three groups, we performed nanopore-based sequencing of full-length 16S amplicon libraries from DNA samples of these feces. The richness of gut microbiota was estimated by the rarefaction curves, which showed similar patterns across samples (Figure 6A). To study alpha diversity in the different EtOH-treated groups, the Shannon index and the observed-OTUs index were calculated. As expected from the antibiotic treatment prior to FMT, the observed-OTUs index was significantly lower in the FMT group, while the relative abundance of OTUs (evenness) was comparable across groups as reflected by the Shannon index (Figure 6B). We then focused on the relative abundance of the top 10 bacterial species identified, which clustered closer female and male + FMT samples according to their predominant *Verrucomicrobia, Firmicutes*, and *Bacteroidetes* phyla (Figure 6C) and revealed a more similar gut microbiota composition at species level between donor female and male + FMT groups compared to baseline male group (Figure 6D). Altogether, these results suggest that FMT has the potential to restore the microbiota composition of male mice into a female-like gut microecosystem more protective to EtOH intoxication (Figure 6E).

## Discussion

Earlier we showed the alterations in the gut-liver axis in response to experimental acute alcohol injury in young rodents (23). Interestingly, changes in the intestinal epithelial barrier associated with increased permeability and modifications in gut microbiota were evident. Importantly, young female mice were more prone to hepatic injury in response to binge drinking, specifically in markers of steatosis and inflammation (23).

In the present study, we focused on extending the current understanding of the effects of ethanol on the gut-liver axis in mature animals. Evidence of impaired intestinal function in aged mice and primates (32, 33), and increase in gut disorders in the aged population has been earlier reported (34).

In this study, we used our previously published model of ethanol intoxication in mature adult mice of 52 weeks of age, and evaluated gut and liver damage, with the hope of understanding EtOH effects on middle-aged population. Our data showed that mature adult male mice displayed increased gut permeability and decreased number of TJs as well as less mucous protection compared with same age female mice, after experimental acute EtOH exposure. Concomitant with our results but obtained in elderly mice, it has been reported that EtOH exposure in aged mice promoted leakage of FITC-dextran into the blood (35), showing intestinal barrier affectation, which in normal conditions is upheld, in part, by TJs proteins such as ZO-1 and occludins (36). It is tempting to speculate that as aging advances, intestinal permeability is higher, and alcohol exerts a more harmful effect in the gut-liver axis. In agreement with this hypothesis, increased gut permeability has been observed in aged mice (33, 37) and older adults (38). To elucidate whether intestinal barrier disruption had a detrimental impact on the liver, we subsequently studied hepatic injury and inflammation. Our results showed that middle-aged male mice are more susceptible to EtOH-induced hepatocellular injury and hepatic lipid accumulation than same age female mice. Of note, our model of acute exposure to EtOH in middle-aged animals confirms previous literature in which chronic exposure to high levels of ethanol leads to hepatic inflammation (6, 39) and aging (35).

Apart from inflammation and hepatocellular injury, aging has been linked with multiple pathophysiological mechanisms, including innate immune responses and alterations in ethanol metabolism (40). Importantly, middle-aged mice were more susceptible to greater liver fibrosis after long-term chronic plus multiple binges (via downregulation of SIRT1) (40).

Alcohol causes dysbiosis thereby disrupting intestinal barrier function, promoting the translocation of microbial LPS into the portal circulation and liver (41). Indeed, it has been demonstrated that alterations in the GVB as decrease in the expression of the adherens junction (AJ) and TJ proteins, and the increase of plasmalemma vesicle-associated protein-1 (PV-1) (42) which is a transmembrane protein associated to the diaphragm of the fenestrated endothelium, is correlated with bacteria dissemination. For instance, *Salmonella* can penetrate the GVB and not only increase ALT levels but also endothelial leakage (43, 44).

Therefore, we hypothesized that fecal microbiota transplantation (FMT) from mature female donors- less susceptible to EtOH intoxication- could attenuate the gut-liver axis impairment observed in same age male mice. Interestingly, our data evidenced that gut microbiota from middle-aged female mice was capable of partially restoring gut permeability, hepatocellular injury and lipid accumulation in same age male mice after EtOH exposure. FMT has been suggested for the management of alcohol-related liver disease (ArLD) by acting on its primary insult, gut dysbiosis. Elegant studies with rodents have suggested that ArLD-related liver injury is transferrable and also treatable by adopting FMT from suitable donors (45). Other publications have shown that FMT between young and aged mice replaced the recipient microbiota composition with a composition resembling the donor, enriched for particular bacterial species, and altered the metabolic potential of the resulting gut microbial composition (46). In agreement with our results, microbes from female donors were established more successfully in the male gastrointestinal tract (47). The gut microbiome is age and sex-specific, affecting inflammation and metabolism in a sex-dependent manner (48). It has been proposed that aging-associated changes in the composition, diversity, and function of gut microbiota increases gut permeability and activates immune responses (49). Moreover, studies revealed that 52 week-old female and male mice show sex differences in intestinal microbiota which could impact morbility and mortality during aging (48). The sexually dimorphic microbiome is called microgenderome (50). Altogether, this set of data evidenced a role for FMT for managing alcohol related disorders in mature adults. Among the bacterial species that showed increased representation in male mice after FMT, *Parabacteroides goldsteinii* stands out, due to its described anti-obesity role, which includes reducing obesity, inducing increased adipose tissue thermogenesis, enhancing intestinal integrity, and reducing levels of inflammation and insulin resistance in mice (51). Another bacterium, *Bacteroides acidifaciens*, also increased in males after FMT and has been associated with the amelioration of liver injury induced by concanavalin-A t via the reduction of hepatocyte apoptosis (52). *Bacteroides acidifaciens* also contributes to acetate production, which protects against the development of non-alcoholic steatohepatitis (NASH) (53). Additionally, *Kineothrix alysoides*, which also showed increased presence in male mice after FMT, demonstrated in a mice model for Metabolic Dysfunction-Associated Steatotic Liver Disease (MASLD) that animals treated with *Kineothrix alysoides* exhibited a reduction in hepatocyte ballooning and fat deposition in the livers of mice fed a high fructose-high fat (HFHF) diet. Moreover, the lipid droplet size in the epididymal fat of HFHF-fed mice with *Kineothrix alysoides* also decreased. These findings indicated that *Kineothrix alysoides* might have the potential to limit fat accumulation caused by the HFHF diet in the liver (54). All these changes in gut microbiota could explain, at least in part, the positive effects that FMT from middle-age female mice donors exerts over middle-age male mice recipients after a protocol of binge drinking.

In our study, we focused on middle age, since it is a very important stage of the life span and strongly impacts health in late life. In fact, the quantity and frequency of binge drinking, has implications for the health and wellbeing of middle-aged adults both concurrently and prospectively. There is a strong need to determine the relationship between alcohol use and health, as well as how factors such as endogenous and exogenous hormones interact with alcohol to influence health in pre- and post-menopausal women.

For an equivalent alcohol intake, women tend to exhibit higher blood alcohol levels than men, despite a faster rate of ethanol elimination. However, the gender disparities in blood alcohol levels following oral intake were not observed after intravenous administration of the dose (55). This gender discrepancy has been linked to lower gastric alcohol dehydrogenase (ADH) activity in women compared to men (56). Interestingly, in individuals over 50 years of age, the Km of gastric ADH activity decreases with age only in men, not in women. Consequently, gender-related differences in first-pass metabolism tend to equalize or even reverse in the elderly, possibly due to gastric mucosal atrophy, which is more prevalent in men than women (56, 57). Further examination of gastric ADH activity in middle-aged female and male mice before and after FMT would provide valuable insights into how gut microbiota may influence alcohol metabolism. Moreover, our experimental work may shed light on potential mechanisms involved in the apparently diverse health effects of binge drinking. The aging phenotype is strongly associated with a state of systemic inflammation, called age-related inflammation (also referred to as inflammaging). Although not all studies report the same direction of change, most studies examining TLR expression in aging mice suggest advanced age leads to a reduction in murine and human TLR expression (58). However, our study was conducted in middled-aged animals of 52 weeks of age and not in 18–24-month-old mice—specifically considered old mice. Therefore, EtOH increased TLR-expression which was ameliorated by FMT. Activation of TLR4 promotes the production of various inflammatory mediators, including a senescence-associated phenotype, clearly reverted after FMT.

In summary, our study reports, for the first time, gender-specific effects of EtOH exposure in mature adult mice in the gastrointestinal system and propose FMT as a novel therapeutic avenue for the reversal of gut leakage, hepatocellular injury and steatosis during aging.

## Data availability statement

The long-read sequencing data of 16S rRNA gene presented in the study are deposited in NCBI Gene Expression Omnibus and are accessible through GEO Series accession number GSE263477.

## Ethics statement

The animal study was approved by the Conserjería de Medio Ambiente, Administración Local y Ordenación del Territorio (PROEX 125.1/20 and 264.2/23). The study was conducted in accordance with the local legislation and institutional requirements.

## Author contributions

AL-P: Data curation, Investigation, Methodology, Writing—original draft, Writing—review & editing. MM: Data curation, Validation, Writing—review & editing, Investigation, Methodology. MG-L: Conceptualization, Data curation, Formal analysis, Investigation, Methodology, Software, Validation, Writing—review & editing. OE-V: Investigation, Methodology, Writing—review & editing. RB-U: Investigation, Methodology, Writing—review & editing. AG: Writing—review & editing, Investigation, Methodology. HH: Investigation, Methodology, Writing—review & editing. HL: Investigation, Methodology, Writing—review & editing. JV: Data curation, Investigation, Methodology, Writing—review & editing. RB: Data curation, Investigation, Methodology, Writing—review & editing. EM-N: Data curation, Funding acquisition, Methodology, Writing—review & editing. SR: Supervision, Validation, Writing—review & editing, Formal analysis, Investigation, Methodology. YN: Funding acquisition, Methodology, Supervision, Writing—original draft, Writing—review & editing. GJ: Data curation, Formal analysis, Funding acquisition, Investigation, Methodology, Supervision, Writing—original draft, Writing—review & editing. FC: Conceptualization, Data curation, Funding acquisition, Project administration, Supervision, Validation, Writing—original draft, Writing—review & editing.

## References

[1] Rehm J Shield KD. Global burden of alcohol use disorders and alcohol liver disease. Biomedicines. (2019) 7:99. 10.3390/biomedicines7040099 31847084 PMC6966598

[2] Cabezas J Bataller R. Alcoholic liver disease: new UK alcohol guidelines and Dry January: enough to give up boozing? Nat Rev Gastroenterol Hepatol. (2016) 13:191–2. 10.1038/nrgastro.2016.39 26956065

[3] Ventura-Cots M Watts AE Bataller R. Binge drinking as a risk factor for advanced alcoholic liver disease. Liver Int. (2017) 37:1281–3. 10.1111/liv.13482 28845617 PMC5656398

[4] Han BH Moore AA Ferris R Palamar JJ. Binge drinking among older adults in the United States, 2015 to 2017. J Am Geriatr Soc. (2019) 67:2139–44. 10.1111/jgs.16071 31364159 PMC6800799

[5] Kepner WE Han BH Nguyen D Han SS Lopez FA Palamar JJ. Past-month binge drinking and cannabis use among middle-aged and older adults in the United States, 2015-2019. Alcohol. (2023) 107:32–7. 10.1016/j.alcohol.2022.07.006 35934163 PMC9933134

[6] Szabo G. Gut-liver axis in alcoholic liver disease. Gastroenterology. (2015) 148:30–6. 10.1053/j.gastro.2014.10.042 25447847 PMC4274189

[7] Gao B Ahmad MF Nagy LE Tsukamoto H. Inflammatory pathways in alcoholic steatohepatitis. J Hepatol. (2019) 70:249–59. 10.1016/j.jhep.2018.10.023 30658726 PMC6361545

[8] Meroni M Longo M Dongiovanni P. Alcohol or gut microbiota: who is the guilty? Int J Mol Sci. (2019) 20. 10.3390/ijms20184568 31540133 PMC6770333

[9] Albillos A De Gottardi A Rescigno M. The gut-liver axis in liver disease: Pathophysiological basis for therapy. J Hepatol. (2020) 72:558–77. 10.1016/j.jhep.2019.10.003 31622696

[10] Diaz Brinton R. Minireview: translational animal models of human menopause: challenges and emerging opportunities. Endocrinology. (2012) 153:3571–8. 10.1210/en.2012-1340 22778227 PMC3404353

[11] Pruett S Tan W Howell GE III Nanduri B. Dosage scaling of alcohol in binge exposure models in mice: An empirical assessment of the relationship between dose, alcohol exposure, and peak blood concentrations in humans and mice. Alcohol. (2020) 89:9–17. 10.1016/j.alcohol.2020.03.011 32259574 PMC8221372

[12] Wang Y Tang J Lv Q Tan Y Dong X Liu H. Establishment and resilience of transplanted gut microbiota in aged mice. iScience. (2022) 25:103654. 10.1016/j.isci.2021.103654 35024588 PMC8733228

[13] Kundu P Lee HU Garcia-Perez I Tay EXY Kim H Faylon LE. Neurogenesis and prolongevity signaling in young germ-free mice transplanted with the gut microbiota of old mice. Sci Transl Med. (2019) 11:aau4760. 10.1126/scitranslmed.aau4760 31723038

[14] D'amato A Di Cesare Mannelli L Lucarini E Man AL Le Gall G Branca JJV. Faecal microbiota transplant from aged donor mice affects spatial learning and memory via modulating hippocampal synaptic plasticity- and neurotransmission-related proteins in young recipients. Microbiome. (2020) 8:140. 10.1186/s40168-020-00914-w 33004079 PMC7532115

[15] Nevzorova YA Bangen JM Hu W Haas U Weiskirchen R Gassler N. Cyclin E1 controls proliferation of hepatic stellate cells and is essential for liver fibrogenesis in mice. Hepatology. (2012) 56:1140–9. 10.1002/hep.25736 22454377 PMC3396430

[16] Nevzorova YA Hu W Cubero FJ Haas U Freimuth J Tacke F. Overexpression of c-myc in hepatocytes promotes activation of hepatic stellate cells and facilitates the onset of liver fibrosis. Biochim Biophys Acta. (2013) 1832:1765–75. 10.1016/j.bbadis.2013.06.001 23770341

[17] Mehlem A Hagberg CE Muhl L Eriksson U Falkevall A. Imaging of neutral lipids by oil red O for analyzing the metabolic status in health and disease. Nat Protoc. (2013) 8:1149–54. 10.1038/nprot.2013.055 23702831

[18] Livak KJ Schmittgen TD. Analysis of relative gene expression data using real-time quantitative PCR and the 2(-Delta Delta C (T)) Method. Methods. (2001) 25:402–8. 10.1006/meth.2001.1262 11846609

[19] Mcmurdie PJ Holmes S. phyloseq: an R package for reproducible interactive analysis and graphics of microbiome census data. PLoS ONE. (2013) 8:e61217. 10.1371/journal.pone.0061217 23630581 PMC3632530

[20] Xu S Zhan L Tang W Wang Q Dai Z Zhou L. MicrobiotaProcess: a comprehensive R package for deep mining microbiome. Innovation. (2023) 4:100388. 10.1016/j.xinn.2023.100388 36895758 PMC9988672

[21] Suzuki T. Regulation of the intestinal barrier by nutrients: the role of tight junctions. Anim Sci J. (2020) 91:e13357. 10.1111/asj.13357 32219956 PMC7187240

[22] Otani T Furuse M. Tight junction structure and function revisited. Trends Cell Biol. (2020) 30:805–17. 10.1016/j.tcb.2020.08.004 32891490

[23] Lamas-Paz A Moran L Peng J Salinas B Lopez-Alcantara N Sydor S. Intestinal epithelial cell-derived extracellular vesicles modulate hepatic injury via the gut-liver axis during acute alcohol injury. Front Pharmacol. (2020) 11:603771. 10.3389/fphar.2020.603771 33408632 PMC7779758

[24] Yao D Dai W Dong M Dai C Wu S. MUC2 and related bacterial factors: therapeutic targets for ulcerative colitis. EBioMedicine. (2021) 74:103751. 10.1016/j.ebiom.2021.103751 34902790 PMC8671112

[25] Chopyk DM Grakoui A. Contribution of the intestinal microbiome and gut barrier to hepatic disorders. Gastroenterology. (2020) 159:849–63. 10.1053/j.gastro.2020.04.077 32569766 PMC7502510

[26] Shojaie L Iorga A Dara L. Cell death in liver diseases: a review. Int J Mol Sci. (2020) 21:9682. 10.3390/ijms21249682 33353156 PMC7766597

[27] Diehl AM Goodman Z Ishak KG. Alcohollike liver disease in nonalcoholics. A clinical and histologic comparison with alcohol-induced liver injury. Gastroenterology. (1988) 95:1056–62. 10.1016/0016-5085(88)90183-7 3410220

[28] Steiner JL Lang CH. Alcohol, adipose tissue and lipid dysregulation. Biomolecules. (2017) 7. 10.3390/biom7010016 28134793 PMC5372719

[29] Jeon S Carr R. Alcohol effects on hepatic lipid metabolism. J Lipid Res. (2020) 61:470–9. 10.1194/jlr.R119000547 32029510 PMC7112138

[30] Hritz I Mandrekar P Velayudham A Catalano D Dolganiuc A Kodys K. The critical role of toll-like receptor (TLR) 4 in alcoholic liver disease is independent of the common TLR adapter MyD88. Hepatology. (2008) 48:1224–31. 10.1002/hep.22470 18792393 PMC7137387

[31] Cai Y Zhou H Zhu Y Sun Q Ji Y Xue A. Elimination of senescent cells by beta-galactosidase-targeted prodrug attenuates inflammation and restores physical function in aged mice. Cell Res. (2020) 30:574–89. 10.1038/s41422-020-0314-9 32341413 PMC7184167

[32] Mitchell EL Davis AT Brass K Dendinger M Barner R Gharaibeh R. Reduced intestinal motility, mucosal barrier function, and inflammation in Aged monkeys. J Nutr Health Aging. (2017) 21:354–61. 10.1007/s12603-016-0725-y 28346561 PMC6057140

[33] Thevaranjan N Puchta A Schulz C Naidoo A Szamosi JC Verschoor CP. Age-associated microbial dysbiosis promotes intestinal permeability, systemic inflammation, and macrophage dysfunction. Cell Host Microbe. (2017) 21:455–66 e454. 10.1016/j.chom.2017.03.002 28407483 PMC5392495

[34] Hall KE Proctor DD Fisher L Rose S. American gastroenterological association future trends committee report: effects of aging of the population on gastroenterology practice, education, and research. Gastroenterology. (2005) 129:1305–38. 10.1053/j.gastro.2005.06.013 16230084

[35] Mcmahan RH Najarro KM Mullen JE Paul MT Orlicky DJ Hulsebus HJ. A novel murine model of multi-day moderate ethanol exposure reveals increased intestinal dysfunction and liver inflammation with age. Immun Ageing. (2021) 18:37. 10.1186/s12979-021-00247-8 34556145 PMC8459518

[36] Lee SH. Intestinal permeability regulation by tight junction: implication on inflammatory bowel diseases. Intest Res. (2015) 13:11–8. 10.5217/ir.2015.13.1.11 25691839 PMC4316216

[37] Brandt A Kromm F Hernandez-Arriaga A Martinez Sanchez I Bozkir HO Staltner R. Cognitive alterations in old mice are associated with intestinal barrier dysfunction and induced toll-like receptor 2 and 4 signaling in different brain regions. Cells. (2023) 12:2153. 10.3390/cells12172153 37681885 PMC10486476

[38] Qi Y Goel R Kim S Richards EM Carter CS Pepine CJ. Intestinal permeability biomarker zonulin is elevated in healthy aging. J Am Med Dir Assoc. (2017) 18:810 e811–4. 10.1016/j.jamda.2017.05.018 28676292 PMC5581307

[39] Benede-Ubieto R Estevez-Vazquez O Guo F Chen C Singh Y Nakaya HI. An experimental DUAL model of advanced liver damage. Hepatol Commun. (2021) 5:1051–68. 10.1002/hep4.1698 34141989 PMC8183170

[40] Ramirez T Li YM Yin S Xu MJ Feng D Zhou Z. Aging aggravates alcoholic liver injury and fibrosis in mice by downregulating sirtuin 1 expression. J Hepatol. (2017) 66:601–9. 10.1016/j.jhep.2016.11.004 27871879 PMC5316497

[41] Liangpunsakul S Toh E Ross RA Heathers LE Chandler K Oshodi A. Quantity of alcohol drinking positively correlates with serum levels of endotoxin and markers of monocyte activation. Sci Rep. (2017) 7:4462. 10.1038/s41598-017-04669-7 28667254 PMC5493657

[42] Brescia P Rescigno M. The gut vascular barrier: a new player in the gut-liver-brain axis. Trends Mol Med. (2021) 27:844–55. 10.1016/j.molmed.2021.06.007 34229973

[43] Spadoni I Zagato E Bertocchi A Paolinelli R Hot E Di Sabatino A. A gut-vascular barrier controls the systemic dissemination of bacteria. Science. (2015) 350:830–4. 10.1126/science.aad0135 26564856

[44] Spadoni I Pietrelli A Pesole G Rescigno M. Gene expression profile of endothelial cells during perturbation of the gut vascular barrier. Gut Microbes. (2016) 7:540–8. 10.1080/19490976.2016.1239681 27723418 PMC5153614

[45] Shasthry SM. Fecal microbiota transplantation in alcohol related liver diseases. Clin Mol Hepatol. (2020) 26:294–301. 10.3350/cmh.2020.0057 32570299 PMC7364360

[46] Parker A Romano S Ansorge R Aboelnour A Le Gall G Savva GM. Fecal microbiota transfer between young and aged mice reverses hallmarks of the aging gut, eye, and brain. Microbiome. (2022) 10:68. 10.1186/s40168-022-01243-w 35501923 PMC9063061

[47] Aburahma A Stewart EL Rana S Larsen R Ward CS Sprague JE. Influence of fecal microbial transplant (FMT) between male and female rats on methamphetamine-induced hyperthermia. Int J Hyperthermia. (2023) 40:2159072. 10.1080/02656736.2022.2159072 36581324

[48] Wang Y Yang H Xu H. Age-specific microbiota in altering host inflammatory and metabolic signaling and metabolome based on sex. Hepatobiliary Surg Nutr. (2022) 11:305–7. 10.21037/hbsn-2022-04 35464278 PMC9023829

[49] Ghosh TS Shanahan F O'toole PW. The gut microbiome as a modulator of healthy ageing. Nat Rev Gastroenterol Hepatol. (2022) 19:565–84. 10.1038/s41575-022-00605-x 35468952 PMC9035980

[50] Vemuri R Sylvia KE Klein SL Forster SC Plebanski M Eri R. The microgenderome revealed: sex differences in bidirectional interactions between the microbiota, hormones, immunity and disease susceptibility. Semin Immunopathol. (2019) 41:265–75. 10.1007/s00281-018-0716-7 30298433 PMC6500089

[51] Wu TR Lin CS Chang CJ Lin TL Martel J Ko YF. Gut commensal Parabacteroides goldsteinii plays a predominant role in the anti-obesity effects of polysaccharides isolated from Hirsutella sinensis. Gut. (2019) 68:248–62. 10.1136/gutjnl-2017-315458 30007918

[52] Wang H Wang Q Yang C Guo M Cui X Jing Z. Bacteroides acidifaciens in the gut plays a protective role against CD95-mediated liver injury. Gut Microbes. (2022) 14:2027853. 10.1080/19490976.2022.2027853 35129072 PMC8820816

[53] Aoki R Onuki M Hattori K Ito M Yamada T Kamikado K. Commensal microbe-derived acetate suppresses NAFLD/NASH development via hepatic FFAR2 signalling in mice. Microbiome. (2021) 9:188. 10.1186/s40168-021-01125-7 34530928 PMC8447789

[54] Choi KJ Yoon MY Kim JE Yoon SS. Gut commensal Kineothrix alysoides mitigates liver dysfunction by restoring lipid metabolism and gut microbial balance. Sci Rep. (2023) 13:14668. 10.1038/s41598-023-41160-y 37674003 PMC10482948

[55] Baraona E Abittan CS Dohmen K Moretti M Pozzato G Chayes ZW. Gender differences in pharmacokinetics of alcohol. Alcohol Clin Exp Res. (2001) 25:502–7. 10.1111/j.1530-0277.2001.tb02242.x 11329488

[56] Pozzato G Moretti M Franzin F Croce LS Lacchin T Benedetti G. Ethanol metabolism and aging: the role of “first pass metabolism” and gastric alcohol dehydrogenase activity. J Gerontol A Biol Sci Med Sci. (1995) 50:B135–41. 10.1093/gerona/50A.3.B135 7743392

[57] Seitz HK Egerer G Simanowski UA Waldherr R Eckey R Agarwal DP. Human gastric alcohol dehydrogenase activity: effect of age, sex, and alcoholism. Gut. (1993) 34:1433–7. 10.1136/gut.34.10.1433 8244116 PMC1374557

[58] Boule LA Kovacs EJ. Alcohol, aging, and innate immunity. J Leukoc Biol. (2017) 102:41–55. 10.1189/jlb.4RU1016-450R 28522597 PMC6608055

