# Supplementary material for: Fecal microbiota transplantation from female donors restores gut permeability and reduces liver injury and inflammation in middle-aged male mice exposed to alcohol
Source: Front Nutr. 2024 Apr 18;11:1393014. doi: 10.3389/fnut.2024.1393014 (PMC11063254; doi:10.3389/fnut.2024.1393014)
Supplement: Supplementary file 1 [file Image_1.pdf]

SUPPLEMENTARY DATA

FECAL MICROBIOTA TRANSPLANTATION FROM FEMALE DONORS RESTORES GUT PERMEABILITY AND PREVENTS LIVER INJURY AND INFLAMMATION IN MIDDLE-AGED MALE MICE EXPOSED TO ALCOHOL

Arantza Lamas-Paz, Mariana Mesquita, Marcos García-Lacarte, Olga Estévez-Vázquez, Raquel Benedé-Ubieto, Alejandro Hionides Gutierrez, Hanghang Wu, Hector Leal Lasalle, Javier Vaquero, Rafael Bañares, Eduardo Martínez-Naves, Sergio Roa, Yulia A. Nevzorova, Gonzalo Jorquera, Francisco Javier Cubero

SUPPLEMENTARY FIGURE LEGENDS

Suppl. Fig. 1. Gut permeability after ethanol (EtOH) intoxication in 52 weeks-old female and male mice. (A) Occludin Immunofluorescence staining of large intestine cryosections in female and male mice after EtOH or phosphate-buffered saline (PBS) oral gavage and **(B)** each quantification. (n= 4). Scale bars: 100 µm. *P<0.05, **P<0.01.

Suppl. Fig. 2. Liver examination after acute alcohol consumption. (A) Macroscopic pictures of the liver of 52-week-old female and male mice after EtOH/PBS oral gavage. **(B)** LW/BW of 52-week-old female and male mice after EtOH/PBS oral gavage. (n=8-15) **(C)** TUNEL staining in liver of 52-week-old female and male mice after EtOH or PBS administration. **(D)** % of TUNEL positive cells after EtOH intoxication in 52-week-old female and male mice. (n=4). Scale bars: 100 µm. ***P<0.001, #P<0.05.

Suppl. Fig. 3. Evaluation of female, male and male + FMT mice after ethanol (EtOH) gavage. (A) Graphical representation of FMT in male mice: Stool from 52-week-old mice was collected and administrated by oral gavage to 52-week-old during 3 days, with a previous antibiotics (ABx) gavage the day before. An EtOH [6 g/kg] oral gavage was administrated at day 18 after FMT and sacrificed 8 h later. **(B)** Macroscopic pictures of liver of 52 weeks-old female, male and male + FMT mice after EtOH oral gavage.

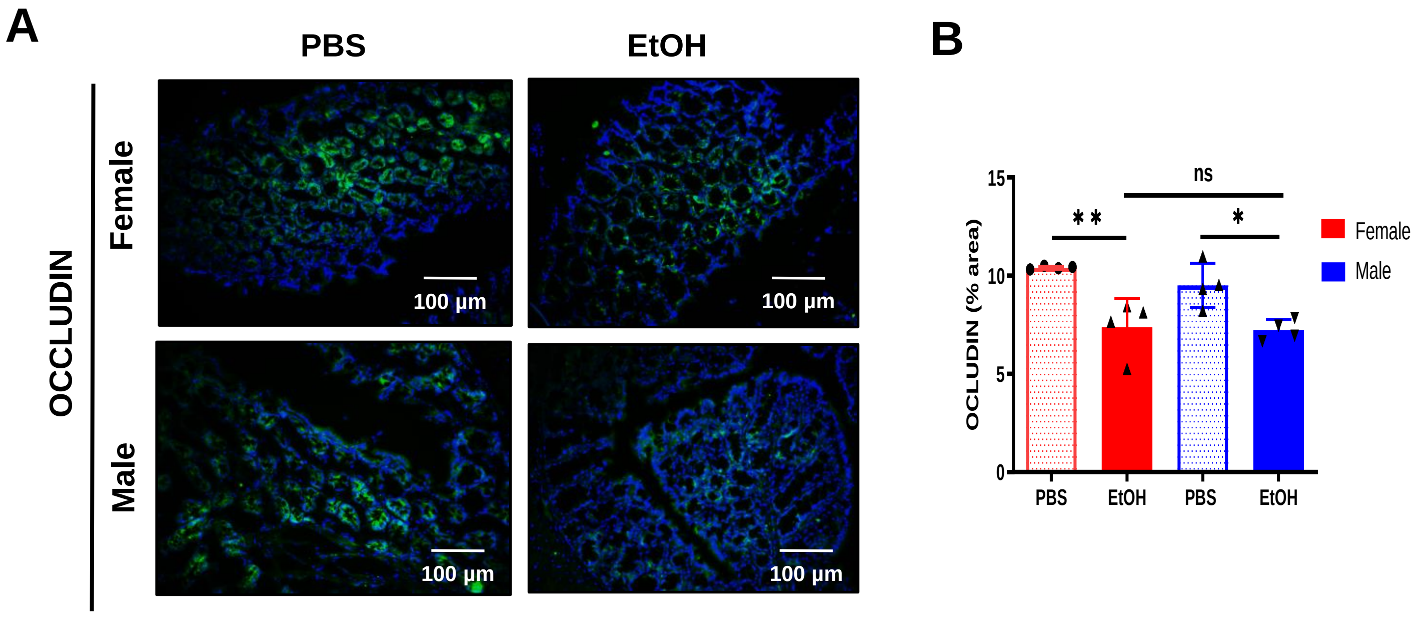
A
PBS
EtOH
OCCLUDIN
Female
Male
100 µm
100 µm
100 µm
100 µm
B
OCLUDIN (% area)
15
10
5
0
ns
**
*
Female
Male
PBS
EtOH
PBS
EtOH

Suppl. Fig. 1

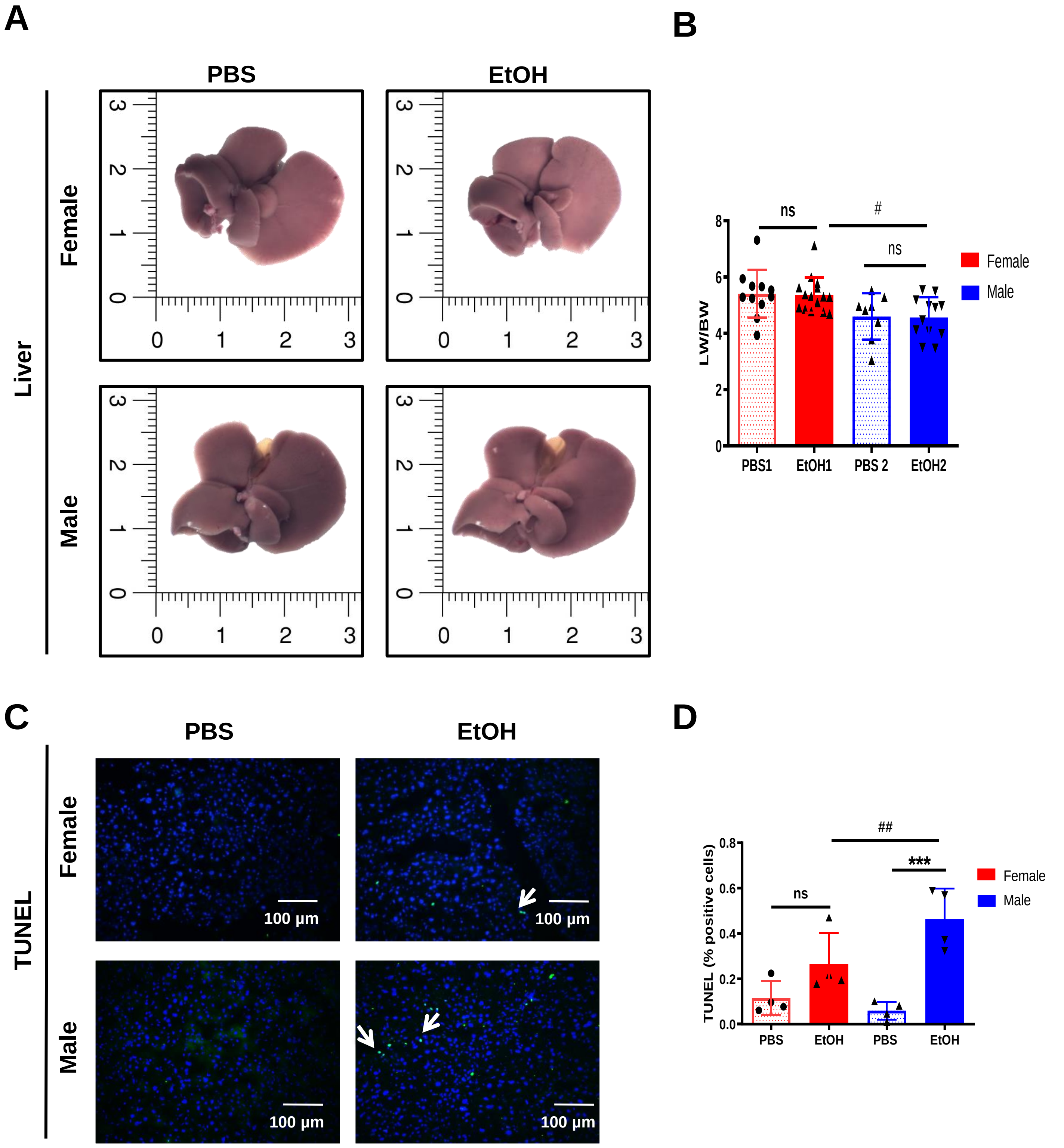
A
PBS
EtOH
Liver
Female
Male
B
LW/BW
ns
#
ns
Female
Male
PBS1
EtOH1
PBS 2
EtOH2
C
PBS
EtOH
TUNEL
Female
Male
100 μm
D
TUNEL (% positive cells)
##
***
ns
Female
Male
PBS
EtOH
PBS
EtOH

Suppl. Fig. 2

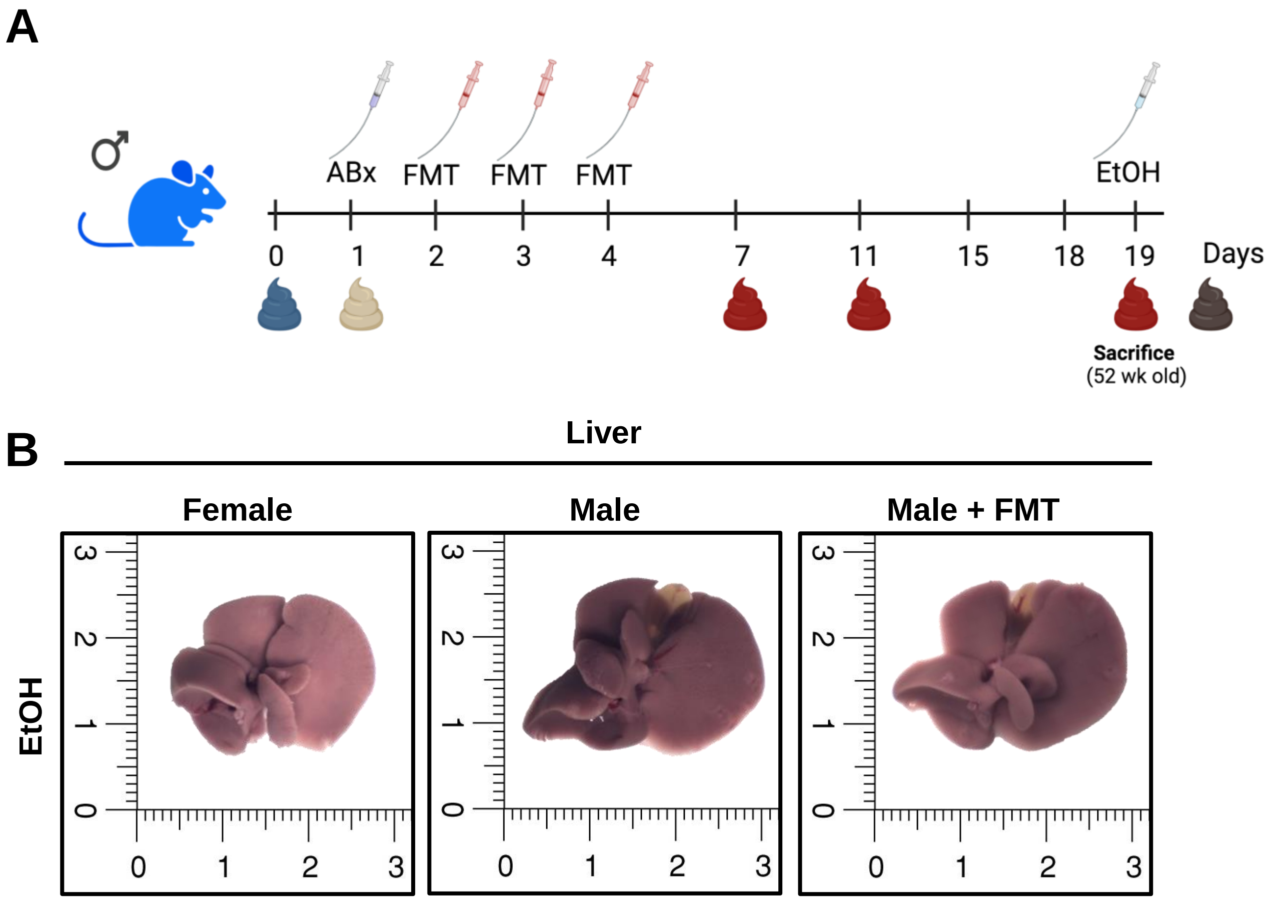
A
ABx
FMT
FMT
FMT
EtOH
0
1
2
3
4
7
11
15
18
19
Days
Sacrifice
(52 wk old)
B
Liver
Female
Male
Male + FMT
EtOH

Suppl. Fig. 3
